# Deep learning-based super-resolution in coherent imaging systems

**DOI:** 10.1038/s41598-019-40554-1

**Published:** 2019-03-08

**Authors:** Tairan Liu, Kevin de Haan, Yair Rivenson, Zhensong Wei, Xin Zeng, Yibo Zhang, Aydogan Ozcan

**Affiliations:** 1 Electrical and Computer Engineering Department, University of California, Los Angeles, CA 90095 USA; 2 Bioengineering Department, University of California, Los Angeles, CA 90095 USA; 3 California NanoSystems Institute (CNSI), University of California, Los Angeles, CA 90095 USA; 4 Department of Surgery, David Geffen School of Medicine, University of California, Los Angeles, CA 90095 USA

## Abstract

We present a deep learning framework based on a generative adversarial network (GAN) to perform super-resolution in coherent imaging systems. We demonstrate that this framework can enhance the resolution of both pixel size-limited and diffraction-limited coherent imaging systems. The capabilities of this approach are experimentally validated by super-resolving complex-valued images acquired using a lensfree on-chip holographic microscope, the resolution of which was pixel size-limited. Using the same GAN-based approach, we also improved the resolution of a lens-based holographic imaging system that was limited in resolution by the numerical aperture of its objective lens. This deep learning-based super-resolution framework can be broadly applied to enhance the space-bandwidth product of coherent imaging systems using image data and convolutional neural networks, and provides a rapid, non-iterative method for solving inverse image reconstruction or enhancement problems in optics.

## Introduction

Coherent imaging systems have many advantages for applications where the specimen’s complex field information is of interest^1^. Since Gabor’s seminal work, various optical and numerical techniques have been suggested^2^ to acquire the complex field of a coherently illuminated specimen. This has allowed for the characterization of absorption and scattering properties of a sample, as well as enabling numerical refocusing at different depths within that sample volume. In order to infer an object’s complex field in a coherent optical imaging system, the “missing phase” needs be retrieved. A classical solution to this missing phase problem is given by off-axis holography^3,4^, which in general results in a reduction of the space-bandwidth product of the imaging system. In-line holographic imaging, which can be used to design compact microscopes^5^, has utilized measurement diversity to generate a set of physical constraints for iterative phase retrieval^6–10^. Recently, deep-learning based holographic image reconstruction techniques have also been demonstrated to create a high-fidelity reconstruction from a single in-line hologram^11–13^, and are capable of further extending the depth-of-field of the reconstructed image^14^.

Several approaches have been demonstrated to improve the resolution of coherent imaging systems^15–20^. Most of these techniques require sequential measurements and assume that the object is quasi-static while a diverse set of measurements are performed on it. These measurements often require the use of additional hardware or sacrifice a degree of freedom such as the sample field-of-view^21^. In recent years, sparsity-based holographic reconstruction methods have also demonstrated that they are capable of increasing the resolution of coherent imaging systems without the need for additional measurements or hardware^22–25^. Sparse signal recovery methods employed in coherent imaging are based on iterative optimization algorithms. These methods usually involve a comprehensive search over a parameter space to obtain the optimal object image and generally result in longer reconstruction times.

Deep learning-based approaches for super-resolution of incoherent microscopy modalities such as brightfield and fluorescence microscopy have also recently emerged^26–30^. These data-driven super-resolution approaches produce a trained deep convolutional neural network that learns to transform low-resolution images into high-resolution images in a single feed-forward (i.e., non-iterative) step. Generative adversarial networks (GANs)^31^ are a form of deep neural network training framework that can be used to ensure that the generated image is sharp and realistic. A GAN is made up of two separate networks. A generator network is used to generate an image that has the same features as the label (ground truth) image, and a discriminator network tries to distinguish between the generated and label (ground truth) images.

In this paper we apply deep learning to enhance the resolution of coherent imaging systems and demonstrate a conditional GAN that is trained to super-resolve both *pixel-limited* and *diffraction-limited* images. Furthermore, we demonstrate the success of this framework on biomedical samples such as thin sections of lung tissue and Papanicolaou (Pap) smear samples. We quantify our results using the structural similarity index (SSIM)^32^ and spatial frequency content of the network’s output images in comparison to the higher resolution images (which constitute our ground truth). This data-driven image super-resolution framework is applicable to enhance the performance of various coherent imaging systems.

## Methods

**Figure 1:**
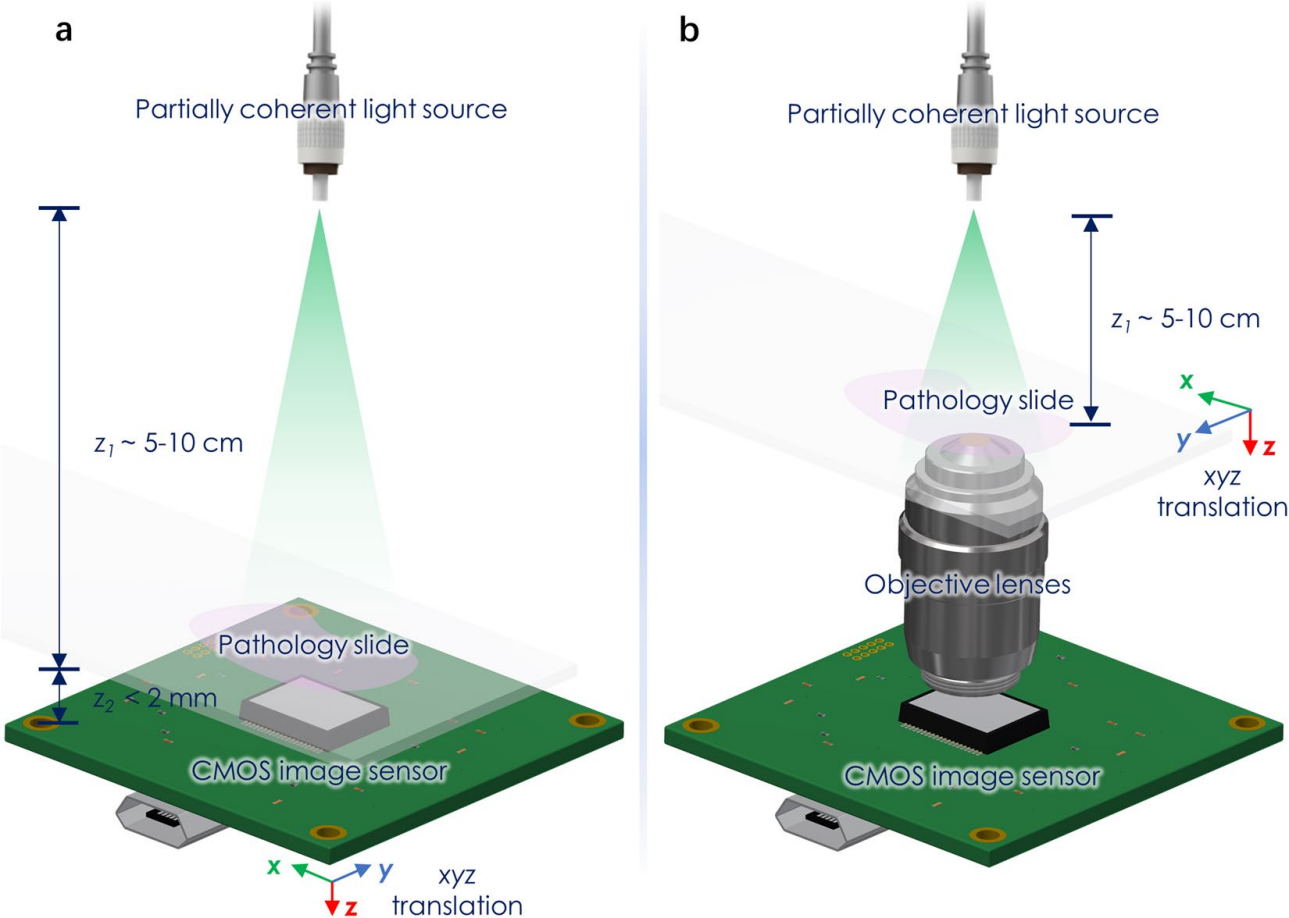
Schematic of the coherent imaging systems. (**a**) A Lens-free on-chip holographic microscope. The sample is placed at a short distance (z_2_ < 2 mm) above the image sensor chip. The resolution of this lensless on-chip imaging modality (without the use of additional degrees of freedom) is pixel size-limited due to its unit magnification. (**b**) A lens-based in-line holographic microscope, implemented by removing the condenser and switching the illumination to a partially-coherent light source on a conventional bright-field microscope. The resolution in this case is limited by the NA of the objective lens.

First, we briefly summarize the methods that we have used in this paper; sub-sequent subsections will provide more information on specific methods employed in our work. We applied the presented deep learning-based super-resolution approach to two separate in-line holographic imaging systems to demonstrate the efficacy of the technique. As illustrated in Fig. 1a,b, the two implemented configurations were a pixel size-limited system (to be referred to as System A) and a diffraction-limited coherent microscopy system (to be referred to as System B). Despite using different methods to create the super-resolved images, as a result of the different image formation models, both of these systems followed similar general hologram reconstruction steps: (1) Raw holograms were collected at different sample to sensor distances, (2) Autofocus was used to determine the accurate sample to sensor distances, (3) Phase was recovered using a multi-height phase recovery algorithm. These steps will be detailed in the following subsections within the Methods^5,7,33–36^.

**Figure 2:**
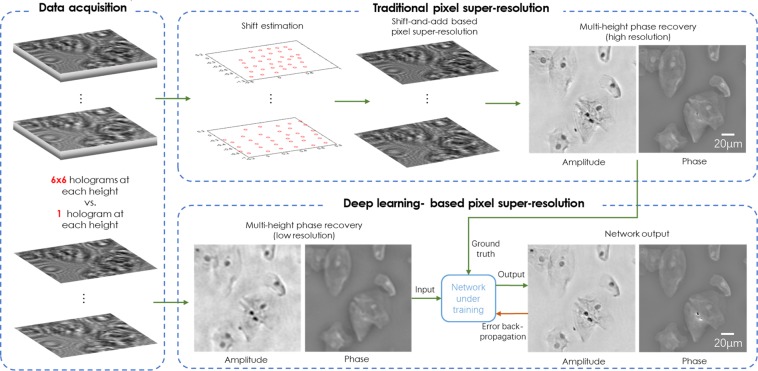
Schematic of the training process for deep-learning based pixel super-resolution. An outline of the data required to generate the network input and ground truth images is shown, together with an overview of how both the traditional super-resolution is performed and how the deep learning super-resolution network is trained.

For the pixel-super-resolution network (System A), the network training process is demonstrated in Fig. 2, which summarizes both the hologram reconstruction procedure as well as the image super-resolving technique with and without using the network. The real and imaginary components of the phase recovered image pairs were used to train the network.

**Figure 3:**
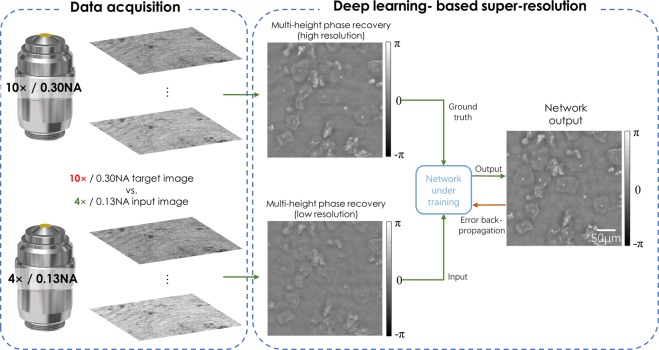
Schematic of the training process for deep learning-based optical super-resolution for an NA-limited coherent imaging system. An outline of the data required to generate the network input and ground truth images is shown, together with an overview of how the deep learning super-resolution network is trained.

For the diffraction-limited super-resolution network (System B), the network training process was demonstrated in Fig. 3. In this case only the phase channel was used to train the network.

### Generation of network input and ground truth super-resolved image labels

For the pixel size-limited coherent imaging system (System A), the super-resolved images were created by collecting multiple low-resolution holograms at different lateral positions, where the CMOS image sensor was sub-pixel shifted by a mechanical stage (MAX606, Thorlabs Inc., Newton, NJ, USA) to create a shift table. Once an accurate shift table was estimated, a shift-and-add based pixel super-resolution algorithm^33^ was applied. The set-up used an illumination wavelength of 550 nm with a bandwidth (Δ*λ*) of ~2 nm (WhiteLase Micro with acousto-optic tunable filter, NKT Photonics, Denmark), a single mode fiber (QPMJ-3S2.5A-488-3.5/125-1-0.3-1, OZ Optics, Canada) with a core diameter of ~3.5 μm and a source-to-sample distance (*z*_1_) of ~5 cm. As a result, the effective spatial coherence diameter at the sensor plane was larger than the width of the CMOS imager chip used in our on-chip imaging system. Therefore, the achievable resolution is limited by the temporal coherence length of the illumination^37^, which is defined as:

1 $${\rm{\Delta }}{{L}}_{c}\approx \sqrt{\frac{2\,\mathrm{ln}\,2}{\pi }}\cdot \frac{{\lambda }^{2}}{n{\rm{\Delta }}\lambda }={\rm{100.47}}\,\mu {\rm{m}}$$

where *n* = 1 is the refractive index. Assuming a sample-to-sensor distance (*z*_2_) of ~300 μm, the effective numerical aperture (NA) of the set-up was limited by the temporal coherence of the source, and is estimated to be:

2 $${\rm{NA}}=n\,\sin \,\theta =n\sqrt{1-{\cos }^{2}\theta }=n\sqrt{1-{(\frac{{z}_{2}}{{z}_{2}+{\rm{\Delta }}{L}_{c}})}^{2}}\approx 0.6624$$

Based on this effective numerical aperture and ignoring the pixel size at the hologram plane, the achievable coherence-limited resolution of our on-chip microscope is approximated as^4^:

3 $$d\propto \frac{\lambda }{{\rm{N}}{\rm{A}}}=\frac{0.55}{0.6624}={\rm{0.8303}}\,\mu {\rm{m}}$$

At the hologram/detector plane, however, the effective pixel pitch of the CMOS image sensor (IMX 081 RGB sensor, Sony Corp., Minato, Tokyo, Japan, pixel size of 1.12 μm) using only one color channel is 2.24 μm. Based on this, the effective pixel size for each super-resolved image after the application of the pixel super-resolution algorithm to 4 raw holograms (2 × 2 lateral positions), 9 raw holograms (3 × 3 lateral positions), and 36 raw holograms (6 × 6 lateral positions) are 1.12 μm, 0.7467 μm and 0.3733 μm, respectively. Based on eq. (3), the effective pixel size achieved by pixel super-resolution using 6 × 6 lateral positions can adequately sample the specimen’s holographic diffraction pattern and is limited by temporal coherence. All of the other images (using 1 × 1, 2 × 2 and 3 × 3 raw holograms) remain pixel-limited in their achievable spatial resolution. This pixel-limited resolution of an on-chip holographic microscope is a result of its unit magnification. This allows the imaging system to have a large imaging field-of-view (FOV) that is only limited by the active area of the opto-electronic image sensor chip. This can easily reach 20–30 mm^2^ and > 10 cm^2^ using state-of-the-art CMOS and CCD imagers, respectively^5^.

For the second set-up (System B), which used lens-based holographic imaging for diffraction-limited coherent microscopy, the low- and high-resolution images were acquired with different objective lenses. For this set-up, the illumination was performed using a fiber coupled laser diode with an illumination wavelength of 532 nm. A 4×/0.13 NA objective lens was used to acquire lower resolution images, achieving a diffraction limited resolution of ~4.09 µm and an effective pixel size of ~1.625 µm. A 10×/0.30 NA objective lens was used to acquire the higher resolution images (ground truth labels), achieving a resolution of 1.773 µm and an effective pixel size of ~0.65 µm.

### Autofocusing and singular value decomposition-based background subtraction

For both types of coherent imaging systems, holograms at 8 different sample-to-sensor distances were collected to perform the multi-height phase recovery^5,7,33–36^. This algorithm requires accurate knowledge of the sample-to-sensor distances used. These were estimated using an autofocusing algorithm. The autofocusing algorithm assigned zero phase to the raw holograms collected by the image sensor and propagated them to different sample to sensor heights using the free space angular spectrum approach^4^. The Tamura of the gradient (ToG) edge sparsity-based criterion was computed^38^ at each height for each hologram and used to determine the corresponding refocusing distance.

For the lens-based diffraction-limited coherent imaging system (System B), the autofocusing algorithm required an additional background subtraction step. For undesired particles or dust associated with the objective lens or other parts of the optical microscope, the diffraction pattern that is formed is independent of the sample and its position. Using this information, a singular value decomposition (SVD)-based background subtraction was performed^39^, after which the ToG-based autofocusing algorithm was successfully applied.

### Multi-height phase recovery

The iterative multi-height phase recovery technique^34^ was applied to eliminate the holographic image artifacts (twin image and self-interference terms^4^) in both of the coherent imaging systems that were used in this work. To perform this, an initial zero-phase was assigned to the intensity/amplitude measurement at the 1st hologram height. Next, the iterative algorithm begins by propagating the complex field to each hologram height until the 8^th^ height is reached, and then backpropagates the resulting fields until the 1^st^ height is reached. While the phase was retained at each hologram height, the amplitude was updated by averaging current amplitude and the square root of the measured intensity at each height.

### Registration between lower resolution and higher resolution (ground truth) images

Image registration plays a key role in generating the training and testing image pairs for the network in both the pixel size-limited and diffraction-limited coherent imaging systems. A pixel-wise registration must be performed to ensure the success of the network in learning the transformation to perform super-resolution.

For both super resolution methods, the low-resolution input images were initially bicubically up-sampled. Following this, a correlation-based registration, which corrected any rotational misalignments or shifts between the images was performed. This registration process correlated the spatial patterns of the phase images and used the correlation to establish an affine transform matrix. This was in turn be applied to the high-resolution images to ensure proper matching of the corresponding fields-of-view between the low-resolution images and their corresponding ground truth labels. Finally, each image was cropped by 50 pixels to each side to accommodate for any relative shift that may have occurred.

For the diffraction-limited coherent imaging system (System B), an additional rough FOV matching step was required before the registration above. For this step, the higher resolution phase images was first stitched together, by calculating the overlap between neighboring images, and using this to stitch them together into a larger image. The corresponding lower resolution phase images are then matched to this larger image. This is done by creating a correlation score matrix between the stitched high resolution image and each of the lower resolution images. Whichever portion of the matrix has the highest correlation score is used to determine which portion of the fused image is cropped out and is used as the input for the network.

### GAN architecture and training process

**Figure 4:**
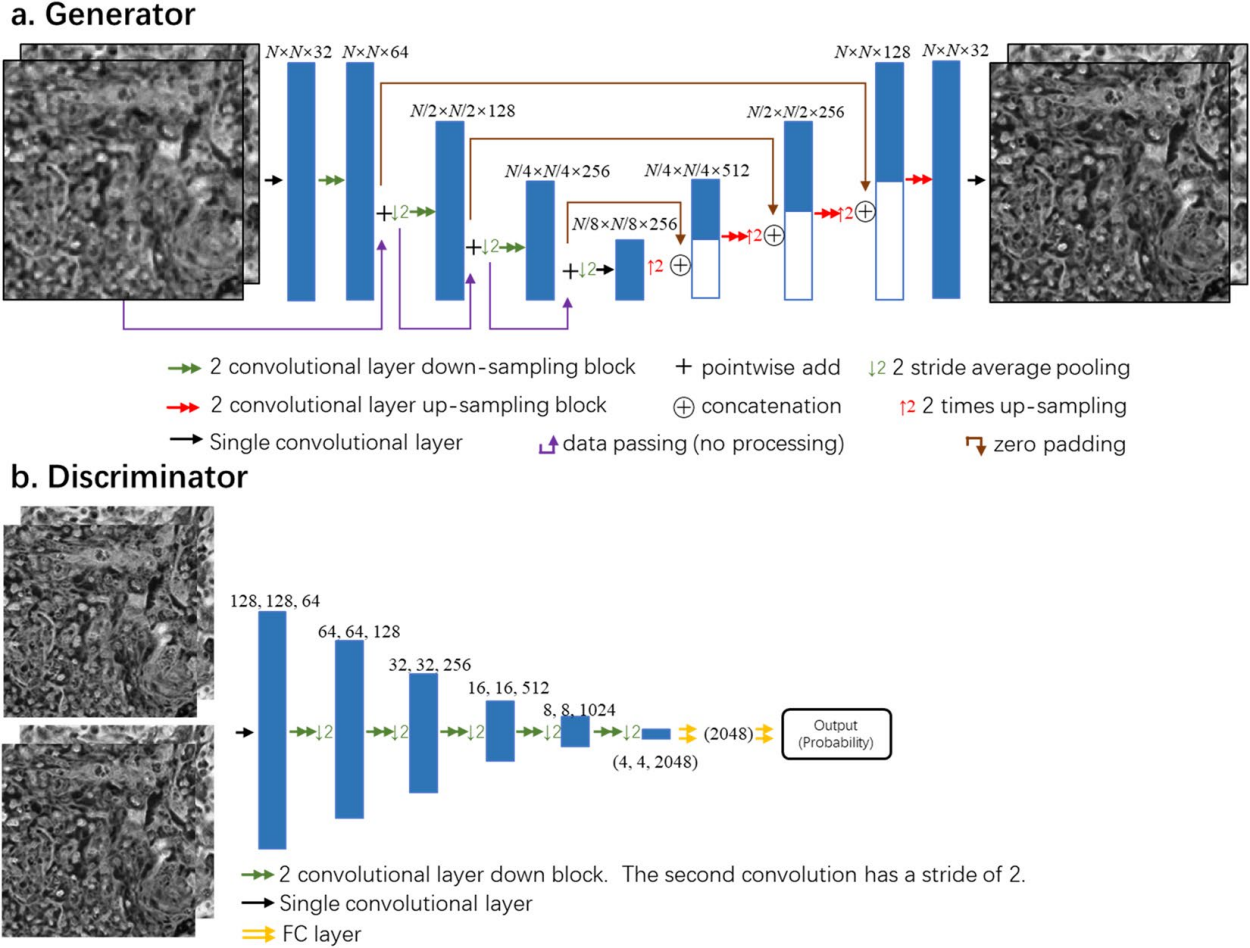
Diagram of the GAN structure. (**a**) Structure of the generator portion of the network. (**b**) Structure of the discriminator portion of the network.

Once the high and low resolution image pairs were accurately registered, they were cropped into smaller image patches (128 × 128 pixels), which were used to train the network. The architectures of the generator (*G*) and the discriminator (*D*) that make up the GAN can be seen in Fig. 4. For both the pixel-size limited and the diffraction-limited coherent imaging systems, the discriminator loss function is defined as:

4 $${l}_{{\rm{d}}{\rm{i}}{\rm{s}}{\rm{c}}{\rm{r}}{\rm{i}}{\rm{m}}{\rm{i}}{\rm{n}}{\rm{a}}{\rm{t}}{\rm{o}}{\rm{r}}}=D{(G({x}_{{\rm{i}}{\rm{n}}{\rm{p}}{\rm{u}}{\rm{t}}}))}^{2}+{(1-D({z}_{{\rm{l}}{\rm{a}}{\rm{b}}{\rm{e}}{\rm{l}}}))}^{2}$$

where *D*(.) and *G*(.) refer to the discriminator and generator network operators, respectively, *x*_*input*_ is the lower resolution input to the generator, and *z*_label_ is the higher resolution label image. For the lensfree holographic imaging system (System A), the generator loss function was defined by:

5 $${l}_{{\rm{g}}{\rm{e}}{\rm{n}}{\rm{e}}{\rm{r}}{\rm{a}}{\rm{t}}{\rm{o}}{\rm{r}}}={L}_{1}\{{z}_{{\rm{l}}{\rm{a}}{\rm{b}}{\rm{e}}{\rm{l}}},G({x}_{{\rm{i}}{\rm{n}}{\rm{p}}{\rm{u}}{\rm{t}}})\}+\gamma \times TV\{G({x}_{{\rm{i}}{\rm{n}}{\rm{p}}{\rm{u}}{\rm{t}}})\}+\alpha \times {(1-D(G({x}_{{\rm{i}}{\rm{n}}{\rm{p}}{\rm{u}}{\rm{t}}})))}^{2}$$

The *L*_1_{*z*_label_,*G*(*x*_input_)} term is calculated using:

6 $${L}_{1}\{{z}_{{\rm{label}}},G({x}_{{\rm{input}}})\}={{\rm{E}}}_{n\_\mathrm{pixels}}({{\rm{E}}}_{n\_\mathrm{channels}}(|G({x}_{{\rm{input}}})-{z}_{{\rm{label}}}|))$$

This finds the absolute difference between each pixel of the generator output image and its corresponding label. E_n_pixels_(.) and E_n_channels_(.) are the expectation values for the pixels with in each image and the channels of each image, respectively. *TV*{*G*(*x*_input_)} represents the total variation loss, which acts as a regularization term, applied to the generator output. Total variation (*TV*) is defined as:

7 $$TV={E}_{n\_\mathrm{channels}}({\sum }_{i,j}|G{({x}_{{\rm{input}}})}_{i+1,j}-G{({x}_{{\rm{input}}})}_{i,j}|+|G{({x}_{{\rm{input}}})}_{i,j+1}-G{({x}_{{\rm{input}}})}_{i,j}|)$$

where the *i* and *j* indices represent the location of the pixels within each channel of the image.

The last term in eq. (5) (i.e., α × (1 − *D*(G(*x*_input_)))^2^) is a function of how well the output image of the generator network can be predicted by the discriminator network. *α* and *γ* are regularization parameters which were set to 0.00275 and 0.015 respectively. As a result of these parameters, the *L*_1_ loss term, *L*_1_{*z*_label_,*G*(*x*_input_)}, made up 60% of the overall loss, while the total variation term, *γ* × *TV*{*G*(*x*_input_)}, was approximately 0.25% of the total loss. The discriminator loss term, *α* × (1 − *D*(*G*(*x*_input_)))^2^, made up the remainder of the overall generator loss. Once the networks were successfully trained, they reach a state of equilibrium where the discriminator network cannot successfully discriminate between the output and label images, and *D*(*G*(*x*_input_)) converged to approximately 0.5.

The loss function for the lens-based coherent microscope images (System B) incorporated an additional structural similarity index (SSIM)^32^ term in addition to the terms included for the lensfree on-chip imaging system, i.e.:

8 $${l}_{{\rm{g}}{\rm{e}}{\rm{n}}{\rm{e}}{\rm{r}}{\rm{a}}{\rm{t}}{\rm{o}}{\rm{r}}}={L}_{1}\{{z}_{{\rm{l}}{\rm{a}}{\rm{b}}{\rm{e}}{\rm{l}}},G({x}_{{\rm{i}}{\rm{n}}{\rm{p}}{\rm{u}}{\rm{t}}})\}+\gamma \times TV\{G({x}_{{\rm{i}}{\rm{n}}{\rm{p}}{\rm{u}}{\rm{t}}})\}+\alpha \times {(1-D(G({x}_{{\rm{i}}{\rm{n}}{\rm{p}}{\rm{u}}{\rm{t}}})))}^{2}\,+\beta \times {\rm{S}}{\rm{S}}{\rm{I}}{\rm{M}}\{G({x}_{{\rm{i}}{\rm{n}}{\rm{p}}{\rm{u}}{\rm{t}}}),{z}_{{\rm{l}}{\rm{a}}{\rm{b}}{\rm{e}}{\rm{l}}}\}$$

*β* is a regularization parameter, set to 0.01, and SSIM{*x*, *z*} is defined as^32^:

9 $${\rm{SSIM}}(x,z)=\frac{(2{\mu }_{x}{\mu }_{z}+{c}_{1})(2{\sigma }_{x,z}+{c}_{2})}{({\mu }_{x}^{2}+{\mu }_{z}^{2}+{c}_{1})({\sigma }_{x}^{2}+{\sigma }_{z}^{2}+{c}_{2})}$$

where *μ*_*x*_, *μ*_*z*_ are the averages of *x*, *z*; ${\sigma }_{x}^{2},\,{\sigma }_{z}^{2}$ are the variances of *x*, *z*, respectively; *σ*_*x,z*_ is the covariance of *x* and *z*; and *c*_1_, *c*_2_ are dummy variables used to stabilize the division with a small denominator. The term *β* × SSIM{*G*(*x*_input_),*z*_label_} was set to make up ~15% of the total generator loss, with the rest of the regularization weights reduced in value accordingly.

Our generator network used an adapted U-net architecture^40^. The network began with a convolutional layer that increased the number of channels to 32 and a leaky rectified linear (LReLU) unit, defined as:

10 $${\rm{L}}{\rm{R}}{\rm{e}}{\rm{L}}{\rm{U}}\,(x)=\{\begin{array}{ll}x & {\rm{for}}\,x > 0\\ 0.1x & {\rm{otherwise}}\end{array}$$

Following this layer, there was a down-sampling and an up-sampling section. Each section consists of three distinct layers, each made up of separate convolution blocks (see Fig. 4a). For the down-sampling section, these residual blocks consisted of two convolution layers with LReLU units acting upon them. At the output of the second convolution of each block the number of channels was doubled. The down-sampling blocks were connected by an average-pooling layer of stride two that down-samples the output of the previous block by a factor of two in both lateral dimensions (see Fig. 4a).

The up-sampling section of the network used a reverse structure to reduce the number of channels and return each channel to its original size. Similar to the down sampling section, each block contained two convolutional layers, each activated by a LReLU layer. At the input of each block, the previous output was up-sampled using a bilinear interpolation and concatenated with the output of the down-sampling path at the same level (see Fig. 4a). Between the two paths, convolutional layer was added to maintain the number of the feature maps from the output of the last residual block to the beginning of the down-sampling path (Fig. 4a). Finally, a convolutional layer was used to reduce the number of output channels to match the size of the label.

The discriminator portion of the network was made up of a convolutional layer, followed by five discriminator blocks, an average pooling layer and two fully connected layers which reduced the output to a single value (see Fig. 4b). Both the label images and the output of the generator network were input into the initial convolutional layer discriminator network. This layer was used to increase the number of channels to 32 and was followed by five discriminator blocks all containing two convolutional layers activated upon by LReLU functions. The first convolution was used to maintain the size of the output, and the second doubled the number of channels while halving the size of the output in each lateral dimension. Next, the average pooling layer was used to find the mean of each channel, reducing the dimensionality to a vector of length 1024 for each patch. Each of these vectors were subsequently fed into two fully connected layers and LReLU activation layers in series. While the first fully connected layer did not change the dimensionality, the second reduced the output of each patch to a single number which was in turn input into a sigmoid function. The output of the sigmoid function represents the probability of the input being either real or fake and was used as part of the generator’s loss function.

The filter size for each convolution was set to be 3 × 3. The trainable parameters are updated using an adaptive moment estimation (Adam)^41^ optimizer with a learning rate 1 × 10^−4^ for the generator network and 1 × 10^−5^ for the discriminator network. The image data were augmented by randomly flipping 50% of the images, and randomly choosing a rotation angle (0, 90, 180, 270 degrees). For each iteration that the discriminator is updated, the generator network is updated four times, which helps the discriminator avoid overfitting to the target images. The convolutional layer weights are initialized using a truncated normal distribution while the network bias terms are initialized to zero. A batch size of 10 is used for the training, and a batch size of 25 is used for validation. The networks chosen for blind testing were those with the lowest validation loss. The number of training steps as well as the training time for each network are reported in Table 1, and the testing times are reported in Table 2.

**Table 1:** Training details for the deep neural networks.

Resolution limiting factor	Tissue type	Low resolution input type	Training dataset size (number of patches before augmentation)	Training time (s)	Number of iterations
Pixel size-limited (System A)	Pap smear	1 × 1 raw hologram	56250	9,078	17,000
	Lung	1 × 1 raw hologram	83700	17,052	28,000
	Lung	2 × 2 raw holograms	83700	9,363	15,000
	Lung	3 × 3 raw holograms	83700	30,480	52,500
Diffraction-limited (System B)	Pap smear	4×/0.13 NA objective lens	65475	46,411	100,000

All the networks were trained with a batch size of 10 using 128 × 128 pixel patches.

**Table 2:** Time for each network to output a 1940 × 1940 pixel image.

Resolution limiting factor	Tissue type	Low resolution input type	Testing Time (s/image)
Pixel size-limited (System A)	Pap smear	1 × 1 raw hologram	1.42
	Lung	1 × 1 raw hologram	1.37
	Lung	2 × 2 raw holograms	1.38
	Lung	3 × 3 raw holograms	1.38
Diffraction-limited (System B)	Pap smear	4×/0.13 NA objective lens	1.26

Each measurement is the average time, calculated using 150 test images.

### Software implementation details

The network was developed using a desktop computer running the Windows 10 operating system. The desktop uses an Nvidia GTX 1080 Ti GPU, a Core i7-7900K CPU running at 3.3 GHz, and 64 GB of RAM. The network was programmed using Python (version 3.6.0) with the TensorFlow library (version 1.7.0).

### Sample preparation

De-identified Pap smear slides were provided by the UCLA Department of Pathology (Institutional Review Board no. 11–003335) using ThinPrep® and SurePath^TM^ preparation. De-identified Hematoxylin and Eosin (H&E) stained human lung tissue slides were acquired from the UCLA Translational Pathology Core Laboratory. We used existing and anonymous specimen, where no subject related information was linked or can be retrieved.

## Results and Discussion

### Super-resolution of a pixel size-limited coherent imaging system (System A)

We first report the performance of the network when applied to the pixel size-limited coherent imaging system using a Pap smear sample and a Masson’s trichrome stained lung tissue section (connected tissue sample). For the Pap smear, two samples from different patients were used for training. For the lung tissue samples, three tissue sections from different patients were used for training. The networks were blindly tested on additional tissue sections from other patients. The FOV of each tissue image was ~20 mm^2^ (corresponding to the sensor active area).

**Figure 5:**
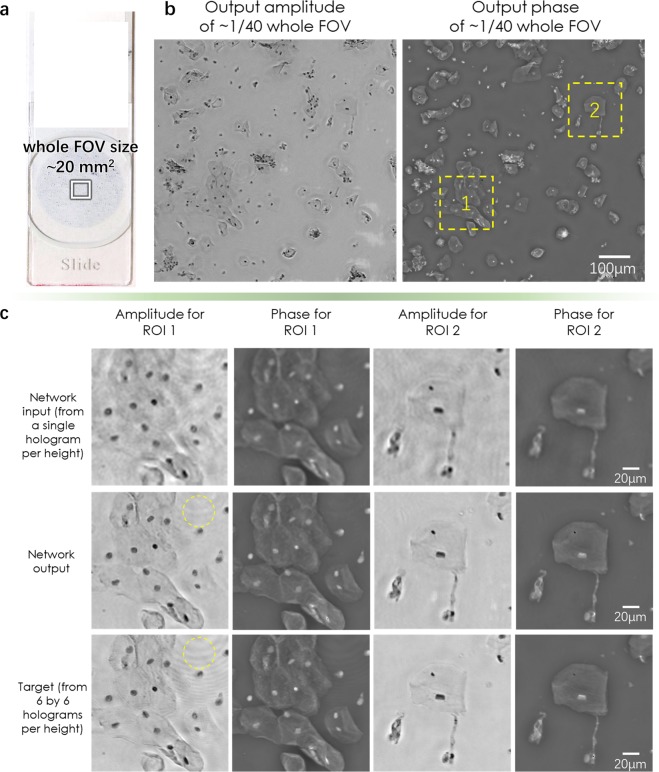
Visualized result for the pixel size-limited system. Deep learning-based pixel super-resolution imaging of a Pap smear slide under 550 nm illumination. (**a**) Whole FOV of the lensfree imaging system. (**b**) Amplitude and phase channels of the network output. (**c**) Further zoom-in of (**b**) for two regions of interest. The marked region in the first column demonstrates the network’s ability to process the artifacts caused by out-of-focus particles within the sample.

**Figure 6:**
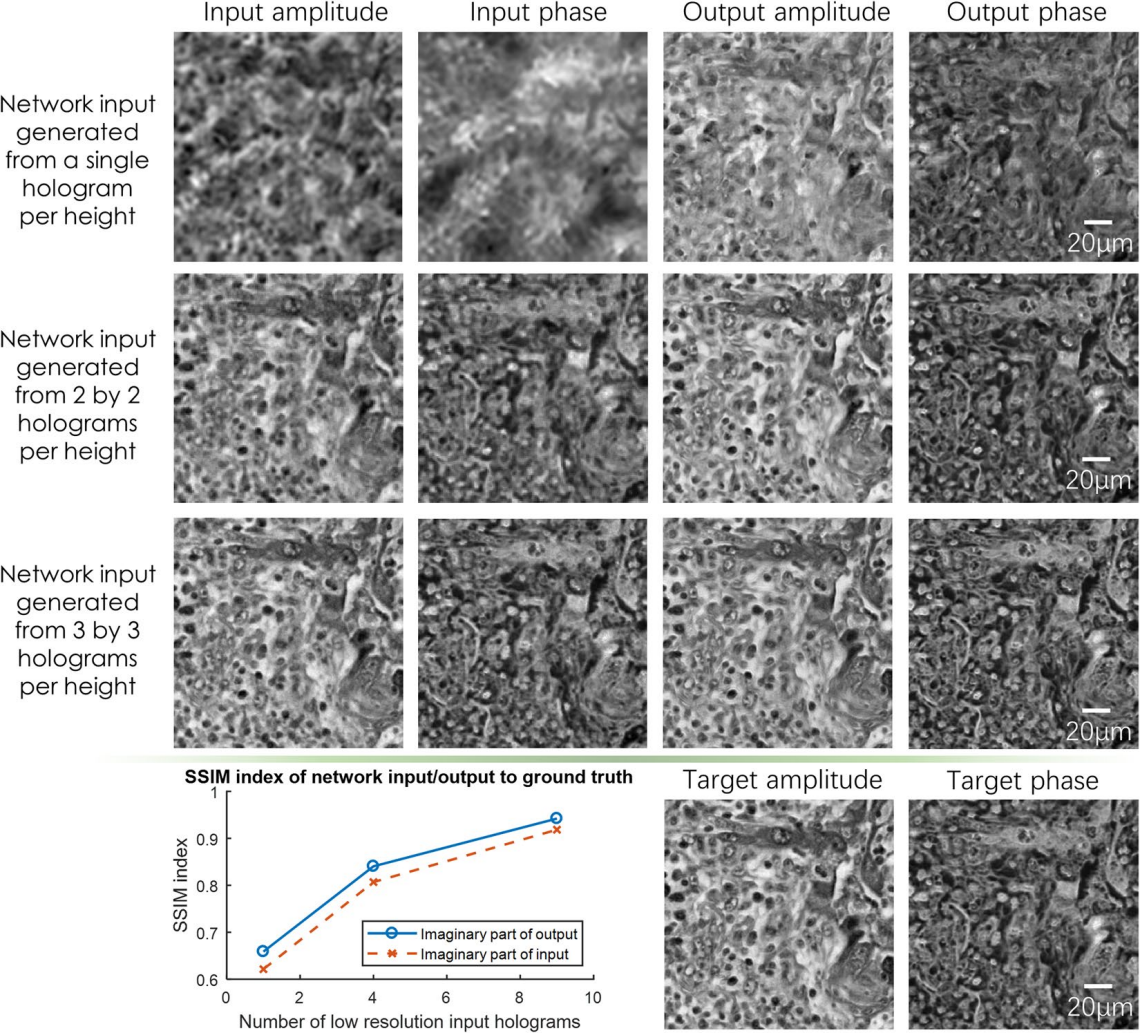
Visualized result for the pixel size-limited system. Comparison of the performances for the deep-learning-based pixel super-resolution methods using different input images. The sample is a Masson’s trichrome stained lung tissue slide, imaged at an illumination wavelength of 550 nm. SSIM values are also shown for the network input and output images for each case. The ground truth (target) image for each SSIM value is acquired using 6 × 6 lensfree holograms per height.

Figure 5 illustrates the network’s super-resolved output images along with pixel-size limited lower resolution input images and the higher resolution ground truth images of a Pap smear sample. The input images have a pixel pitch of 2.24 µm, and the label images have an effective pixel size of 0.37 µm (see the Methods section). For lung tissue sections, we proved the efficacy of our super-resolution technique (Fig. 6) using three different deep networks, where the input images for each network used a different pixel size (2.24 µm, 1.12 µm, and 0.7467 µm, corresponding to 1 × 1, 2 × 2 and 3 × 3 lateral shifts, respectively, as detailed in the Methods section). Unlike the less densely connected Pap smear sample results, the network output is missing some of the spatial details that are seen by the high-resolution images of the lung tissue imaging when the input pixel size is at its coarsest level (2.24 µm pixel size). These spatial features are recovered by the other two networks that use smaller input pixels as shown in Fig. 6.

We also report the SSIM values with respect to the reference label images in order to further evaluate the performance of our network output when applied to a pixel size-limited coherent imaging system. The average SSIM values for the entire image FOV (~20 mm^2^) are listed in Table 3, where the input SSIM values were calculated between the bicubic interpolated lower resolution input images and the ground truth images. The results clearly demonstrate the improved structural similarity of the network output images.

**Table 3:** Average SSIM values for the lung and Pap smear samples for the deep neural network output (also see Figs 5 and 6 for sample images in each category).

Resolution limiting factor	Tissue type	Low resolution input type	Input SSIM		Output SSIM	
			Imaginary	Real	Imaginary	Real
Pixel size-limited	Pap smear	1 × 1 raw hologram	0.9097	0.9135	0.9392	0.9442
	Lung	1 × 1 raw hologram	0.6213	0.5404	0.6587	0.7135
	Lung	2 × 2 raw holograms	0.8069	0.8205	0.8405	0.8438
	Lung	3 × 3 raw holograms	0.9185	0.9184	0.9422	0.9347

**Figure 7:**
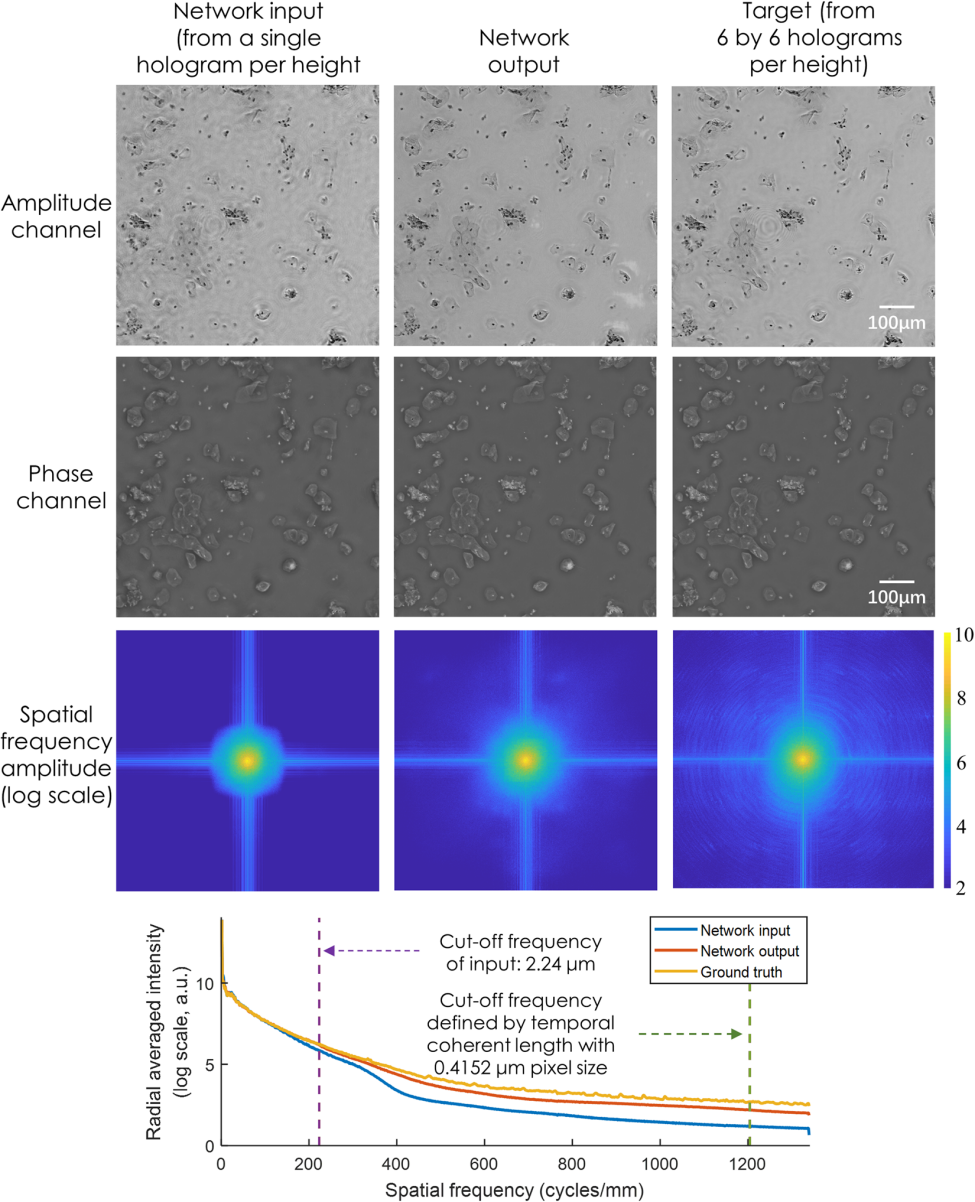
Spatial frequency analysis for the pixel size-limited system. Radially-averaged spatial frequency spectra of the network input, network output and target images, corresponding to a lensfree on-chip coherent imaging system.

In addition to SSIM, we also report the improved performance of our network output using spatial frequency analysis. Figure 7 reports the 2-D spatial frequency spectra and the associated radially-averaged frequency intensity of the network input, network output and the ground truth images corresponding to our lensfree on-chip imaging system. Another indication that the super-resolution is successful is that the higher spatial frequency components in the output of the network are very close to the spatial frequencies of the ground truth image.

### Super-resolution of a diffraction-limited coherent imaging system (System B)

**Figure 8:**
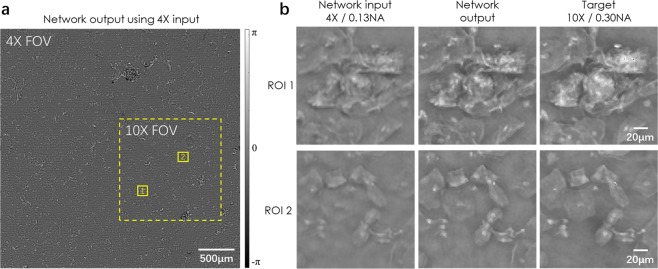
Visualized result for the diffraction-limited system. Deep learning-based super-resolution imaging of a Pap smear slide under 532 nm illumination using a lens-based holographic microscope. (**a**) Phase channel of the network output image. (**b**) Zoomed-in images of (**a**).

**Figure 9:**
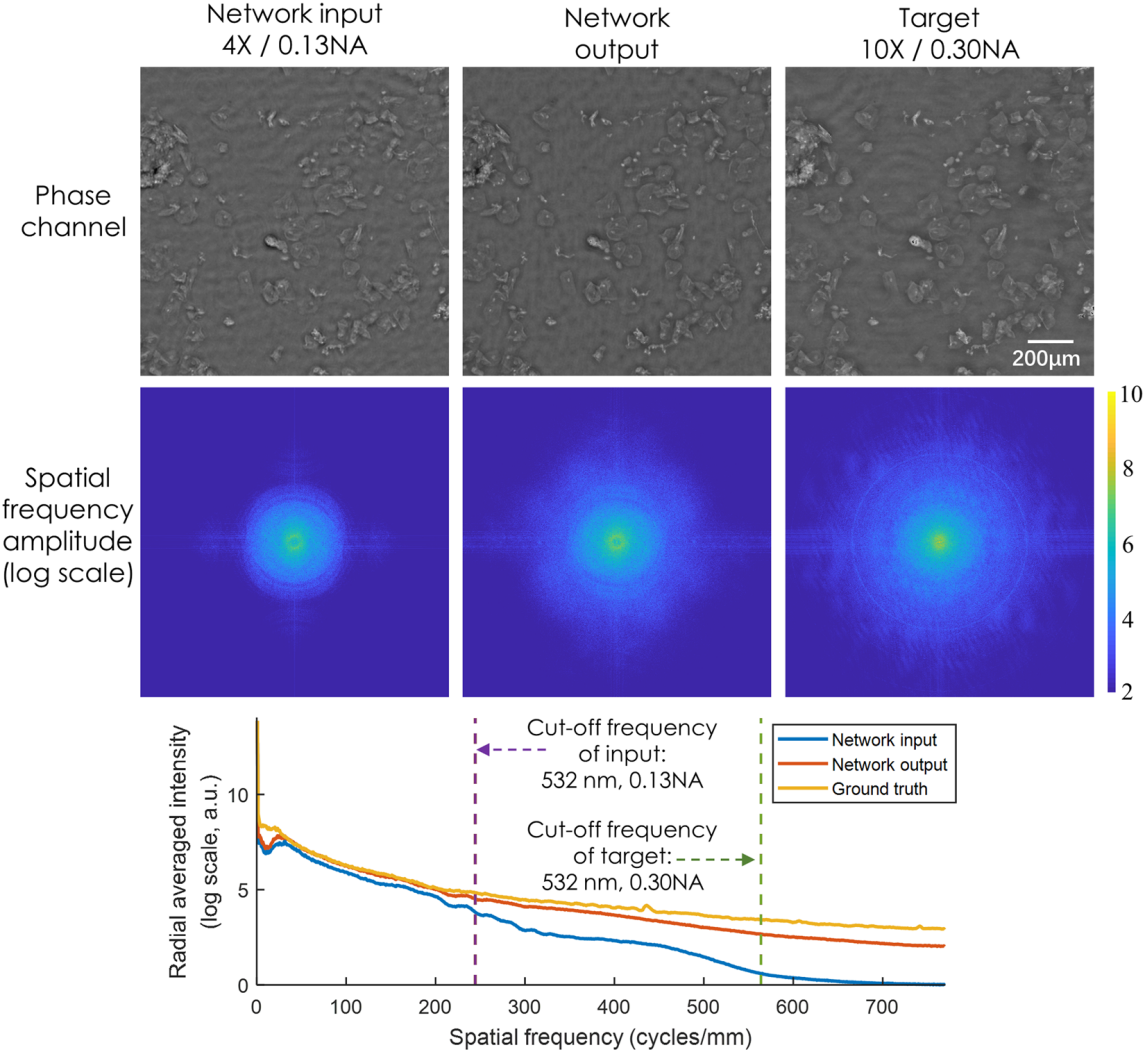
Spatial frequency analysis for the diffraction-limited system. Radially-averaged spatial frequency spectra of the network input, network output and target images, corresponding to a lens-based coherent imaging system.

To demonstrate that this super-resolution framework can also be applied to a diffraction-limited coherent imaging system, we trained another network using the same architecture (see the Methods section) with images taken from a Pap smear sample. As in the pixel super-resolution case reported earlier, two samples were obtained from two different patients, and the trained network was blindly tested on a third sample obtained from a third patient. The input images were obtained using a 4×/0.13 NA objective lens and the reference ground truth images were obtained by using a 10×/0.30 NA objective lens. Figure 8 illustrates a visual comparison of the network input, output and label images, providing the same conclusions as in Figs 5 and 6. Similar to the pixel size-limited coherent imaging system, we analyzed the performance of our network using spatial frequency analysis, the results of which are reported in Fig. 9. As in Fig. 7, the higher spatial frequencies of the network output image approach the spatial frequencies observed in the ground truth images.

The SSIM values for this system do not reveal as large of a trend as was observed for the lensfree on-chip microscopy system reported earlier. They only show a very small increase from a value of 0.876 for the input image to 0.879 for the network output. This is mainly due to increased coherence related artifacts and noise, compared to the lensfree on-chip imaging set-up. The lens-based design has several optical components and surfaces within the optical beam path, making it susceptible to coherence induced background noise and related image artifacts, which can affect the SSIM calculations.

## Conclusion

We have presented a GAN-based framework that can super-resolve images taken using both pixel size limited and diffraction limited coherent imaging systems. The framework was demonstrated on biologically connected thin tissue sections (lung and Pap smear samples) and the results were quantified using structural similarity index and spatial frequency spectra analysis. The proposed framework provides a highly optimized, non-iterative reconstruction engine that rapidly performs resolution enhancement, without the need for any additional parameter optimization.

The proposed approach is not restricted to a specific coherent imaging modality and is broadly applicable to various coherent image formation techniques. The starting point for our super-resolution technique is after the phase recovery step, which can be obtained by means of e.g., in-line, off-axis, phase-shifting holography, among other approaches. Since it has been proven to work for both pixel size-limited and diffraction-limited imaging systems, it will be applicable to other coherent systems that have similar resolution limitations. One of the techniques that will highly benefit from the proposed framework is off-axis holography. The proposed technique might be used to bridge the space-bandwidth-product gap between off-axis and in-line coherent imaging systems, while retaining the single-shot and high sensitivity advantages of off-axis image acquisition systems.

## Data Availability

Manuscript related data can be requested from the corresponding author.
